# Evaluation of risk factors for cytomegalovirus DNAemia after end of regular prophylaxis after heart transplantation

**DOI:** 10.1002/iid3.1075

**Published:** 2023-11-17

**Authors:** Moritz Benjamin Immohr, Daniel Oehler, Freya Sophie Jenkins, Nikolas Kalampokas, Vincent Hendrik Hettlich, Dennis Sigetti, Fabian Voß, Hannan Dalyanoglu, Hug Aubin, Payam Akhyari, Artur Lichtenberg, Udo Boeken

**Affiliations:** ^1^ Department of Cardiac Surgery Medical Faculty and University Hospital Düsseldorf, Heinrich‐Heine‐University Düsseldorf Düsseldorf Germany; ^2^ Department of Cardiac Surgery, Medical Faculty RWTH Aachen University Aachen Germany; ^3^ Division of Cardiology, Pulmonology and Angiology Medical Faculty and University Hospital Düsseldorf, Heinrich‐Heine‐University Düsseldorf Düsseldorf Germany

**Keywords:** cardiac allograft vasculopathy, cytomegalovirus, heart transplantation, prophylaxis

## Abstract

**Background:**

Cytomegalovirus (CMV) infections after heart transplantation (HTx) can cause cardiac allograft vasculopathy. Consequently, monitoring and prophylaxis for cytomegalovirus deoxyribonucleic acid (CMV‐DNAemia) within the first weeks after HTx is recommended.

**Methods:**

All patients who underwent HTx between September 2010 and 2021 surviving the first 90 days (*n* = 196) were retrospectively reviewed. The patients were divided on the prevalence of CMV‐DNAemia during the first postoperative year after the end of the prophylaxis. A total of *n* = 35 (20.1%) developed CMV‐DNAemia (CMV group) and were compared to patients without CMV‐DNAemia (controls, *n* = 139). The remaining patients (*n* = 22) were excluded due to incomplete data.

**Results:**

Positive donors and negative recipients (D+/R−) and negative donors and positive recipients (D−/R+) serology was significantly increased and D−/R− decreased in the CMV group (*p* < .01). Furthermore, the mean age was 57.7 ± 8.7 years but only 53.6 ± 10.0 years for controls (*p* = .03). Additionally, the intensive care unit (*p* = .02) and total hospital stay (*p* = .03) after HTx were approximately 50% longer. Interestingly, the incidence of CMV‐DNAemia during prophylaxis was only numerically increased in the CMV group (5.7%, respectively, 0.7%, *p* = .10), the same effect was also observed for postoperative infections. Multivariate analyses confirmed that D+/R− and D−/R+ CMV immunoglobulin G match were independent risk factors for postprophylaxis CMV‐DNAemia.

**Conclusion:**

Our data should raise awareness of CMV‐DNAemia after the termination of regular prophylaxis, as this affects one in five HTx patients. Especially old recipients as well as D+/R− and D−/R+ serology share an elevated risk of late CMV‐DNAemia. For these patients, prolongation, or repetition of CMV prophylaxis, including antiviral drugs and CMV immunoglobulins, may be considered.

## Graphical abstract

Prophylaxis for cytomegalovirus (CMV) infections is recommended after heart transplantation as it can cause cardiac allograft vasculopathy. About 20% of patients developed CMV‐DNAemia within the first postoperative year after end of the regular 90‐day prophylaxis. Especially old recipients as well as positve donor and negative recipients and negative donor and positive recipients CMV serology share an elevated risk for late CMV‐DNAemia and therefore prolongation or repeptition of prophlyaxis should be considered.

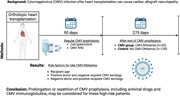

## 1 INTRODUCTION

Within the last decades, tremendous progress has been achieved in postoperative survival after heart transplantation (HTx).^1^ However, the development of cardiac allograft vasculopathy (CAV) continues to limit the long‐term outcome after HTx, as it represents the main cause of long‐term graft dysfunction.^2^, ^3^, ^4^ Although the development of CAV is multifactorial and not fully understood, it is also associated with cytomegalovirus (CMV) infections in the postoperative course.^2^, ^3^, ^4^ In this context, the term cytomegalovirus deoxyribonucleic acid (CMV‐DNAemia) is often used to represent any kind of detected symptomatic or asymptomatic replication of CMV in the patient's blood.^5^

CMV is a commonly observed herpes virus that can occur in immunosuppressed patients due to either primary infection, reinfection, or reactivation.^5^ Therefore, the serology of pretransplant CMV immunoglobulin G (IgG) of both the donor and the recipient has been reported as an important risk factor, especially in the case of transplants from CMV IgG positive donors for CMV IgG negative recipients.^6^, ^7^ Consequently, pharmacological prophylaxis that includes antiviral drugs or intravenous CMV immunoglobulins (CMV‐IVIG) is often administered during the first 3–6 months after HTx.^6^, ^8^ However, the ideal duration of prophylaxis and the risk of early recurrence of CMV‐DNAemia after withdrawal of the prophylaxis remain unclear.^9^, ^10^, ^11^

As CMV‐DNAemia is associated with CAV that limits long‐term survival after HTx, our objective was to analyze the incidence of early CMV‐DNAemia after the end of regular postoperative CMV prophylaxes. Furthermore, we aimed to evaluate risk factors for CMV‐DNAemia to identify patients with increased risk who may benefit from prolonged or repetitive prophylaxis schemes.

## 2 METHODS

### 2.1 Patients and study design

**Figure 1:**
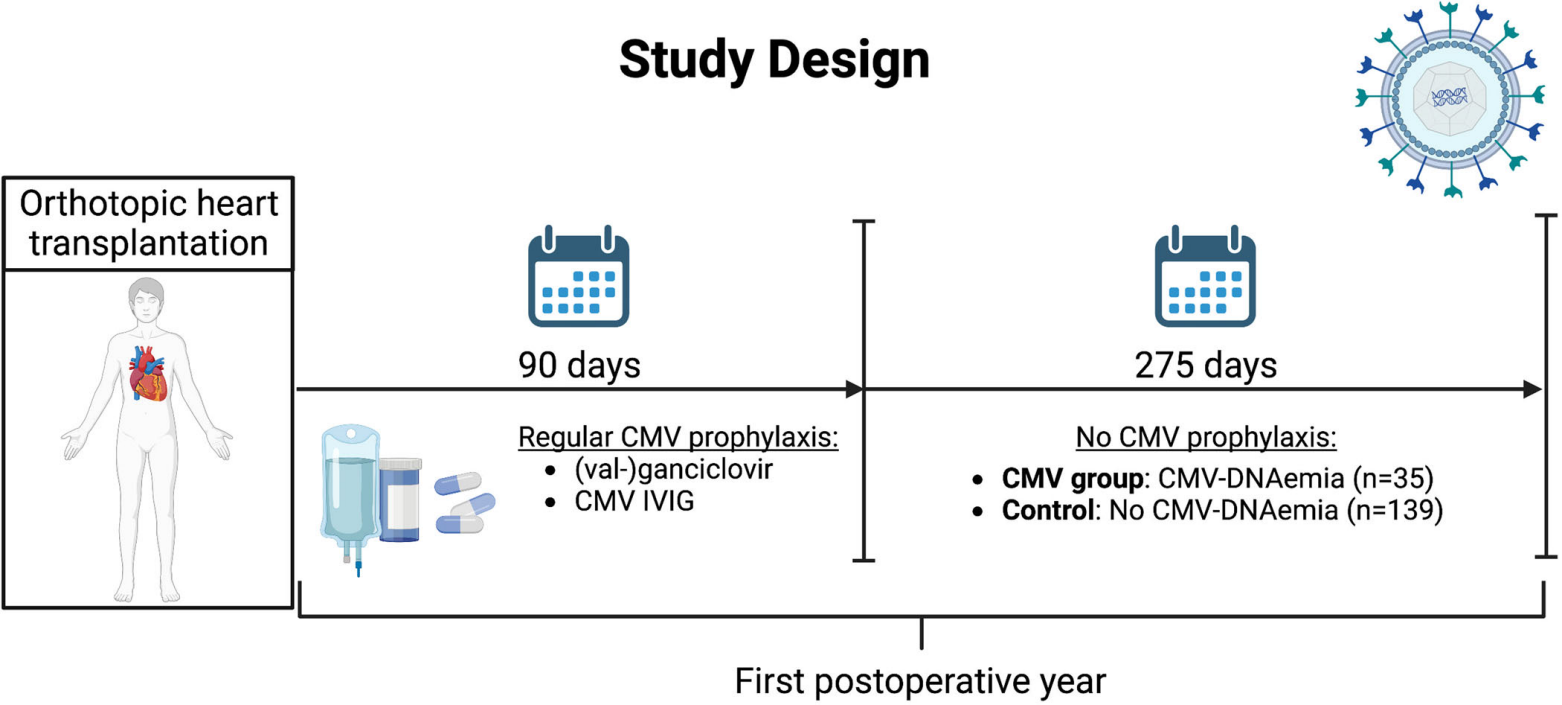
Schematic illustration of the study protocol. CMV, cytomegalovirus; CMV‐DNAemia, CMV deoxyribonucleic acid; CMV IVIG, intravenous CMV immunoglobulins.

For this retrospective observational study, all adult patients who underwent HTx between 2010 and 2021 in a single center were reviewed (*n* = 231). The study objectives were to evaluate the incidence of early CMV reactivation after the end of regular CMV prophylaxis after HTx, as well as to identify potential risk factors. Therefore, we focus on the short‐term occurrence of CMV‐DNAemia instead of the associated long‐term effects on donor grafts such as CAV. Patients who survived the first 90 days after surgery and had completed at least 1 year of follow‐up were included in the study (*n* = 196). The patients were assigned to two different study groups regarding the appearance of CMV‐DNAemia after the end of the regular institutional 90‐day prophylaxis scheme within the first postoperative year, as shown in Figure 1. In 20.1% of the included patients, CMV‐DNAemia was detected (CMV group, *n* = 35). These patients were compared with patients without observed CMV‐DNAemia (control, *n* = 139) to identify possible risk factors for CMV‐DNAemia after the end of regular 90‐day CMV prophylaxis. All reported patients had survived the initial 90 days postoperatively and therefore had completed and ended regular CMV prophylaxis. The follow‐up was then continued until the end of the first postoperative year and CMV‐DNAemia was examined by polymerase chain reaction (PCR) test of the patients' blood at least every 3 months or whenever the patient was hospitalized or CMV‐DNAemia was suspected by clinical evidence.

### 2.2 Surgical procedure and perioperative management

The patients were transplanted orthotopically with a biatrial or bicaval technique. After organ recovery, all donor hearts were preserved in a cold storage solution without a specialized organ preservation system for transport. The period between the removal of the donor organ from cold storage and the release of the aortic cross‐clamp was defined as warm ischemia. Primary graft dysfunction was defined following the current classification of the International Society for Heart and Lung Transplantation. For immunosuppression, a protocol consisting of a pharmacological triple therapy that included tacrolimus (target level: 9–12 ng/mL), mycophenolate mofetil (target level: 1.5–4.0 µg/dL), and prednisolone was followed. No induction therapy was used for any of the patients. For potential rejection of grafts endomyocardial biopsies were performed, and where appropriate, treated with high‐dose prednisolone, immunoadsorption, plasmapheresis, anti‐T‐lymphocyte IgG, and intravenous IgM‐enriched human immunoglobulin.

### 2.3 CMV prophylaxis, monitoring, and therapy scheme

All reported patients followed a standardized institutional CMV prophylaxis scheme.^8^ Therefore, the CMV IgG serology of donors and recipients, kidney function, leukocyte count, and potential concomitant treatment for rejection of recipients' grafts were used for risk stratification. After transplantation of grafts from CMV IgG positive donors (D+) as well as from CMV IgG positive recipients (R+) (including D+/R+, D+/R−, and D−/R+ but not D−/R− serology), recipients were postoperatively prophylactically treated with 5 mg/kg bodyweight ganciclovir per day since the second postoperative day, respectively, 900 mg of valganciclovir for a total of 90 days. In the event of impaired kidney function, the dose of (val‐)ganciclovir was adjusted (glomerular filtration rate [GFR] = 30–60 mL/min: 2.5 mg ganciclovir/day, respectively, 450 mg valganciclovir/day; GFR < 30 mL/min: 1.25 mg ganciclovir/day, respectively, 450 mg of valganciclovir every other day; hemodialysis: 100 mg of valganciclovir per day). In patients with a D+/R− CMV IgG constellation as well as in patients with a GFR < 25 mL/min hyper CMV‐IVIG (Cytotect CP; Biotest AG) were also administered during the first 3 consecutive postoperative days (bodyweight < 75 kg: 50 mL CMV‐IVIG; bodyweight ≥ 75 kg: 100 mL CMV‐IVIG). Furthermore, patients with leucopenia (white blood cells < 2.5×10^9^/L) and patients who were treated for acute organ rejection also regularly underwent CMV‐IVIG application. During the initial hospital stay, CMV‐PCR was performed twice a week to examine potential CMV‐DNAemia. Subsequently, PCR was performed once a week for the first 3 months postoperatively. Between the third and sixth postoperative months, CMV‐DNAemia was tested every other week and then once a month until the end of the first postoperative year. From then on, PCR was performed every 3 months.

In the case of CMV‐DNAemia, patients with less than 1000 CMV copies/mL were treated with 1800 mg of valganciclovir per day. Patients with >1000 CMV copies/mL, all symptomatic cases, and patients with impaired kidney function (GFR < 25 mL/min) received 5 mg/kg body weight intravenous ganciclovir twice a day plus 50 mL, respectively, 100 mL of CMV‐IVIG per day for three consecutive days.

### 2.4 Statistics

Statistical tests were performed using SPSS Statistics version 28.0.1.1 (IBM Corporation). All results are displayed as mean values with the standard deviation, respectively, percentage of the whole. The variables were compared using nonparametric two‐tailed Mann–Whitney *U* tests for continuous ones or Fisher's exact tests for categorial parameters as the Gaussian distribution was not assumed due to the unbalanced group sizes. To identify potential risk factors for CMV‐DNAemia after prophylaxis termination, a binary linear logistic regression was used for multivariate analysis. For this purpose, parameters with a maximum *p* = .10 of the univariate analysis were evaluated with respect to their proposed clinical relevance.

## 3 RESULTS

### 3.1 Recipient age may increase the risk of CMV‐DNAemia

Table 1 shows the preoperative recipient data for the patients. Neither concomitant diseases such as diabetes, chronic kidney injury, or obstructive pulmonary disease nor preoperative laboratory values including recipients' CMV IgG and IgM serology differed between patients with and without detected CMV‐DNAemia after the end of the prophylaxis scheme. Additionally, preoperative mechanically circulatory support was also comparable between groups. However, univariate analyses revealed a significantly older recipient age of the CMV group (57.7 ± 8.7 years) compared to controls (53.6 ± 11.0 years) indicating a potential association.

**Table 1:** Preoperative recipient parameters.

Recipient variables	CMV group (*n* = 35)	Control (*n* = 139)	*p* Value
Age, year (SD)	57.7 (8.7)	53.6 (11.0)	.03
Female gender, *n* (%)	6 (17.1)	41 (29.5)	.20
Height, cm (SD)	174 (9)	174 (9)	.84
Weight, kg (SD)	79.7 (11.2)	77.9 (16.4)	.40
Body mass index, kg/m² (SD)	26.3 (3.5)	25.5 (4.8)	.18
High urgency waitlist status, *n* (%)	17 (48.6)	59 (42.4)	.57
Etiology			.43
Ischemic cardiomyopathy, *n* (%)	17 (48.6)	54 (38.8)	
Dilated cardiomyopathy, *n* (%)	16 (45.7)	73 (52.5)	
Other, *n* (%)	2 (5.7)	12 (8.6)	
Ventricular assist device, *n* (%)	19 (54.3)	64 (46.0)	.45
Extracorporeal life support, *n* (%)	0 (0.0)	6 (4.3)	.60
Concomitant diseases			
Diabetes mellitus, *n* (%)	6/34 (17.6)	28 (20.1)	>.99
Hemodialysis, *n* (%)	1 (2.9)	7 (5.1)	>.99
Arterial hypertension, *n* (%)	25/34 (73.5)	80 (57.6)	.12
Pulmonary hypertension, *n* (%)	2/34 (5.9)	13 (9.4)	.74
Chronic obstructive pulmonary disease, *n* (%)	3/34 (8.8)	13 (9.4)	>.99
Mechanical ventilation, *n* (%)	0/34 (0.0)	9 (6.5)	.21
Laboratory values			
CMV IgG positive, *n* (%)	24 (68.6)	85 (61.2)	.44
CMV IgM positive, *n* (%)	0 (0.0)	5 (3.6)	.58
Hemoglobin, g/dL (SD)	12.0 (2.4)	11.9 (2.1)	.90
Creatinine, mg/dL (SD)	1.31 (0.62)	1.41 (1.13)	.85
Glomerular filtration rate, mL/min (SD)	66.8 (24.3)	66.5 (27.0)	.76
Bilirubin, mg/dL (SD)	0.7 (0.5)	0.8 (0.8)	.95
Aspartate aminotransferase, U/L (SD)	38.0 (34.9)	37.2 (60.0)	.36
Lactate dehydrogenase, U/L (SD)	382 (566)	316 (217)	.30

*Note*: Patients were assigned into two different study groups regarding the prevalence of detected cytomegalovirus deoxyribonucleic acid in the patient's blood (CMV‐DNAemia) after the end of the prophylaxis during the first postoperative year after orthotopic heart transplantation. Patients with CMV‐DNAemia, *n* = 35 were compared to control patients without CMV‐DNAemia (control), *n* = 139. In case of missing data about a patient for a parameter, the total number of analyzed patients is marked in the corresponding row.

Abbreviations: CMV, cytomegalovirus; IgG, immunoglobulin G; IgM, immunoglobulin M; SD, standard deviation.

### 3.2 Impact of donor parameters on the risk for CMV‐DNAemia

Table 2 shows the corresponding donor data as listed in the Eurotransplant donor report before organ recovery. Contrary to recipient parameters, the age of donors was not associated with increased risk of CMV risk (*p* = .75). However, there was a statistical trend toward an increased incidence of diabetes (CMV group: 37.5%, control: 13.0%, *p* = .06) and drug abuse (CMV group: 25.9%, control: 9.8%, *p* = .05) of the CMV group's donors. Furthermore, we observed a slightly decreased reported left ventricular ejection fraction of the grafts from the CMV group before recovery (57.0 ± 9.8%, respectively, 62.1 ± 9.5, *p* = .04). However, clinical relevance remains questionable. Meanwhile, the CMV serology also did not differ between the two groups.

**Table 2:** Donor parameters.

Donor variables	CMV group (*n* = 35)	Control (*n* = 139)	*p* Value
Age, year (SD)	41.9 (11.7)	42.5 (12.6)	.75
Female gender, *n* (%)	13 (37.1)	65 (46.8)	.35
Height, cm (SD)	176 (8)	175 (8)	.31
Weight, kg (SD)	83.8 (17.3)	78.3 (12.8)	.17
Body mass index, kg/m^2^ (SD)	27.8 (7.4)	25.6 (3.6)	.34
Cardiopulmonary resuscitation, *n* (%)	13 (37.1)	35 (25.2)	.20
Ejection fraction, % (SD)	57.0 (9.8)	62.1 (9.5)	.04
Concomitant diseases			
Arterial hypertension, *n* (%)	11/20 (55.0)	34/67 (50.7)	.80
Diabetes mellitus, *n* (%)	6/16 (37.5)	6/46 (13.0)	.06
Smoking, *n* (%)	23/31 (74.2)	72/117 (61.5)	.21
Drug abuse, *n* (%)	7/27 (25.9)	11/112 (9.8)	.05
Alcohol, *n* (%)	13/27 (48.1)	49/114 (43.0)	.67
Laboratory values			
CMV IgG positive, *n* (%)	18 (51.4)	63 (45.3)	.57
CMV IgM positive, *n* (%)	1/12 (8.3)	2/44 (4.5)	.52
Hemoglobin, g/dL (SD)	10.6 (2.9)	10.1 (2.5)	.33
Lactate dehydrogenase, U/L (SD)	421 (303)	493 (593)	.79

*Note*: Donor parameters as listed in the Eurotransplant donor report. Patients were assigned into two different study groups regarding the prevalence of detected cytomegalovirus deoxyribonucleic acid in the patient's blood (CMV‐DNAemia) after the end of the prophylaxis during the first postoperative year after orthotopic heart transplantation. Patients with CMV‐DNAemia, *n* = 35 were compared to control patients without CMV‐DNAemia (control), *n* = 139. In case of missing data about a patient for a parameter, the total number of analyzed patients is marked in the corresponding row.

Abbreviations: CMV, cytomegalovirus; IgG, immunoglobulin G; IgM, immunoglobulin M; SD, standard deviation.

### 3.3 Donor and recipient CMV serology affects the incidence of CMV‐DNAemia

Table 3 shows the intraoperative and postoperative parameters, including details of the allocation procedure. While the predicted mismatch in the heart mass ratio did not affect the incidence of CMV‐DNAemia after the end of prophylaxis, there were multiple effects associated with the CMV IgG serology. On one hand, the matching of positive donors and negative recipients (D+/R−) and of negative donors and positive recipients (D−/R+) was associated numerically with increased risk (CMV group: 25.7%, control: 12.2%, respectively, CMV group: 42.9%, control: 28.1%). On the other hand, D−/R− constellation was associated with significantly less observed cases (CMV group: 5.7%, control: 26.6%). For D+/R+, no differences were observed.

**Table 3:** Donor and recipient allocation and operative outcome.

Outcome variables	CMV group (*n* = 35)	Control (*n* = 139)	*p* Value
Donor and recipient allocation			
Predicted heart mass ratio, % (SD)	13.5 (9.0)	12.5 (10.8)	.31
Cytomegalovirus IgG matching			
Donor positive/recipient positive	9 (25.7)	46 (33.1)	.54
Donor positive/recipient negative	9 (25.7)	17 (12.2)	.06
Donor negative/recipient positive	15 (42.9)	39 (28.1)	.10
Donor negative/recipient negative	2 (5.7)	37 (26.6)	<.01
Intraoperative parameters			
Total graft ischemic time, min (SD)	218 (35)	214 (51)	.73
Transport time, min (SD)	156 (39)	149 (49)	.24
Warm ischemia, min (SD)	61.5 (12.2)	65.8 (14.6)	.07
Total operative time, min (SD)	414 (107)	422 (108)	.99
Extracorporeal circulation, min (SD)	249 (61)	250 (68)	.99
Postoperative outcome			
Primary graft dysfunction			
Duration of catecholamine therapy			
Dobutamine, h (SD)	102 (85)	90 (53)	.89
Epinephrine, h (SD)	130 (99)	116 (74)	.68
Norepinephrine, h (SD)	205 (218)	139 (186)	.03
va‐ECMO, *n* (%)	10 (28.6)	27 (19.4)	.25
Support duration, day (SD)	8.7 (6.9)	6.1 (4.8)	.25
Deceased on support, *n* (%)	0 (0.0)	2 (7.4)	>.99
Postoperative morbidity			
Infective complications, *n* (%)	11 (31.4)	25 (18.0)	.10
In‐hospital CMV‐DNAemia, *n* (%)	2 (5.7)	1 (0.7)	.10
Acute graft rejection, *n* (%)	3 (8.6)	11 (7.9)	>.99
Hemodialysis on ICU, *n* (%)	22/33 (66.7)	69/132 (52.3)	.17
Neurological events, *n* (%)	6 (17.1)	17 (12.2)	.42
Rethoracotomy, *n* (%)	6 (17.1)	40 (28.8)	.20
Postoperative hospital stay, d (SD)	64.1 (54.2)	44.3 (28.6)	.03
Postoperative ICU and IMC stay, d (SD)	32.2 (31.5)	21.6 (24.2)	.02
Mechanical ventilation, h (SD)	166 (203)	109 (154)	.25
Postoperative 1‐year survival	33 (94.3)	128 (92.1)	>.99

*Note*: Operative outcome after heart transplantation. Patients were assigned into two different study groups regarding the prevalence of detected cytomegalovirus deoxyribonucleic acid in the patient's blood (CMV‐DNAemia) after the end of the prophylaxis during the first postoperative year after orthotopic heart transplantation. Patients with CMV‐DNAemia, *n* = 35 were compared to control patients without CMV‐DNAemia (control), *n* = 139. In case of missing data about a patient for a parameter, the total number of analyzed patients is marked in the corresponding row.

Abbreviations: ICU, intensive care unit; IgG, immunoglobulin G; IMC, intermediate care; SD, standard deviation; va‐ECMO, venoarterial extracorporeal life support.

### 3.4 Postoperative intensive care and hospital stay increase the risk for CMV‐DNAemia

There were no differences between the groups regarding the duration of graft ischemia and operation time, as well as primary graft dysfunction and perioperative adverse events such as acute kidney failure, acute graft rejection or neurological events. Considering postoperative infections and CMV‐DNAemia within the initial HTx hospital stay, only a numerically increased incidence for the CMV group (infections: 31.4%, in‐hospital CMV‐DNAemia: 5.7%) compared to the control (infections: 18.0%, in‐hospital CMV‐DNAemia: 0.7%) was observed. However, patients in the CMV group remained about 50% longer in the hospital (61.1 ± 54.2 days, respectively, 44.3 ± 28.6 days, *p* = .03) and in the intensive and intermediate care unit (32.2 ± 31.5 days, respectively, 21.6 ± 24.2 days, *p* = .02) after the HTx procedure.

### 3.5 D+/R− and D−/R+ CMV IgG serology are confirmed risk factors in a multivariate analysis

Multivariate analysis was performed to evaluate independent risk factors for CMV‐DNAemia after the end of the prophylaxis (Table 4). The multivariate model consisted of the parameter recipient age, postoperative hospital stay, postoperative intensive and intermediate care stay, in‐hospital CMV‐DNAemia, as well as D+/R− and D−/R+ CMV IgG match. Binary linear regression confirmed both D+/R− and D−/R+ CMV IgG mismatch as independent and strong risk factors (odds ratio: 6.24, respectively, 2.78) for CMV‐DNAemia after termination of the prophylaxis. The older recipient age was only associated with a small effect (odds ratio: 1.05, *p* = .05). the remaining three parameters were not associated with an increased risk of CMV‐DNAemia.

**Table 4:** Multivariate analysis.

Parameter	Regression coefficient	Standard error	*p* Value	Odds ratio
Recipient age	0.05	0.02	.05	1.05
Postoperative hospital stay	0.01	0.01	.12	1.01
Postoperative ICU and IMC stay	<0.01	0.01	.77	1.00
In‐hospital CMV‐DNAemia	1.75	1.36	.20	5.74
CMV IgG D+/R−	1.83	0.59	<.01	6.24
CMV IgG D−/R+	1.02	0.47	.03	2.78

*Note*: Multivariate analysis of risk factors for CMV‐DNAemia in the patient's blood after the end of the prophylaxis during the first postoperative year after orthotopic heart transplantation.

Abbreviations: CMV, cytomegalovirus; CMV‐DNAemia, cytomegalovirus deoxyribonucleic acid; ICU, intensive care unit; IgG, immunoglobulin G; IMC, intermediate care.

## 4 DISCUSSION

CMV‐DNAemia remains a serious complication after HTx as it is associated with CAV. Therefore, prophylaxis is commonly applied within the first months after HTx. However, we observed early CMV‐DNAemia after the end of regular prophylaxis in about one in five patients. Therefore, we evaluated our patient cohort for possible risk factors for early CMV‐DNAemia after the end of regular prophylaxis. Multivariate analysis confirmed that CMV IgG negative patients receiving organs from CMV IgG positive donors, but also CMV IgG positive recipients with negative donors, are at high risk for CMV‐DNAemia. Furthermore, the univariate analysis indicated an increased risk for older recipients, as well as recipients with prolonged postoperative intensive care and hospital stay. For these patients, prolongation or repetition of the prophylaxis may be considered to minimize the risk of CMV‐related CAV.

Due to the association of DMV‐DNAemia with the development of CAV, in general prophylaxis or preemptive therapy is performed regularly after HTx.^6^, ^11^ Although the good efficacy of our previously reported institutional prophylaxis scheme with val‐/ganciclovir and additional CMV‐IVIG for high‐risk patients has been confirmed, we later observed early CMV‐DNAemia in about 20% of patients.^8^ In line with this, Gupta et al. reported a high incidence of CMV‐DNAemia and even CMV disease within 3 months after the end of a comparable prophylaxis for HTx patients in a case series of high‐risk D+/R− CMV IgG match.^9^ Furthermore, another study reported an incidence of 29% of CMV disease in HTx patients with D+/R− CMV IgG match within approximately 1 year after val‐/ganciclovir prophylaxis.^12^ Finally, a large American multicenter study by Santos et al. covering more than 2200 HTx patients reported a significantly increased incidence of CMV disease after the first postoperative 100 days compared to the proposed time frame with ongoing prophylaxis and was able to correlate these CMV infections with increased mortality in the following course.^10^

Univariate analyses indicated a correlation between increased recipient age and a prolonged postoperative course with early CMV‐DNAemia after the end of prophylaxis in our cohort. The association between recipient age and CMV‐DNAemia has already been described by other groups.^13^, ^14^ Additionally, the association with a prolonged and complicated postoperative course in these patients, who are more complex and are likely to be critically ill, due to reactivation of CMV, also seems very likely.^15^, ^16^, ^17^ The constellation of D+/R− CMV IgG is considered an important risk factor for postoperative CMV‐DNAemia.^6^, ^7^, ^11^, ^18^ Consequently, we could confirm a numerical increase in the incidence of D+/R− CMV IgG serology in the CMV group. Furthermore, we observed an even higher incidence of D−/R+ CMV IgG serology, which was prevalent in the CMV group in approximately 43% of cases. In these patients, reactivation of CMV seems to be the most possible explanation.^6^ Unlike D+/R−, they were not regularly treated with additional CMV‐IVIG during prophylaxis, which could be an explanation.^8^ CMV‐IVIG has been reported to be an effective adjuvant for the prevention of posttransplant CMV‐DNAemia, especially in high‐risk patients.^8^, ^19^, ^20^ Furthermore, as expected, the match of D−/R− CMV IgG was associated with a significantly reduced risk of CMV DNAemia in our cohort, although these patients, according to current recommendations, did not receive antiviral prophylaxis regularly after HTx.^6^

To confirm the relevant risk factors for the development of early CMV‐DNAemia, we then performed an additional multivariate analysis that included the parameters of CMV serology, prolonged postoperative course, and increased recipient age described above. The latter two could not be confirmed as a significant risk factor for CVM‐DNAemia after the end of the prophylaxis. Additionally, regardless of the *p* value, the odds ratio indicated only a narrow clinical impact in our cohort. As mentioned above, recipient age has been reported as a risk factor for CMV‐DNAemia in the literature.^13^, ^14^ However, in line with our results, Mendez‐Eirin et al. reported a hazard ratio = 1.02 (95% confidence interval: 1.00–1.1) for recipient age indicating comparable clinical effects as in our cohort.^13^ On the contrary, D+/R− and D−/R+ CMV IgG serology were independent and important risk factors for CMV‐DNAemia in our multivariate model, which confirms both the literature about the general risk of CMV‐DNAemia after HTx, as well as the few available case series focusing on CMV‐DNAemia after the end of regular prophylaxis in particular.^6^, ^7^, ^8^, ^9^, ^10^, ^11^, ^12^, ^18^

Our study has its limitations: Although the underlying data were collected prospectively throughout the study period, the study represents a retrospective observational design and is therefore limited in certain ways. A priori power analysis for the estimation of adequate sample sizes could not be performed, and the groups are rather small. Additionally, the single‐center design further strengthens this effect. Furthermore, we focused on the early period after the end of prophylaxis. Large multicenter data with long‐term follow‐up would be beneficial to examine longer‐term effects of late CMV‐DNAemia after HTx as well as to correlate CMV‐DNAemia after the end of the prophylaxis with possible development of CAV. In addition, evaluating CMV cell‐mediated immunity by modified enzyme‐linked immunospot assay makes it possible to directly measure the immune response of patients to CMV and is therefore a useful tool for further research projects in the future. However, we were able to report first insights and name significant risk factors for the occurrence of CMV‐DNAemia in the early period after the end of prophylaxis, which may help to improve the therapy.

Approximately 20% of HTx patients suffer from early CMV‐DNAemia after the end of regular prophylaxis. As CMV‐DNAemia can cause CAV, our data should raise awareness. Although the study is limited and the results are preliminary, we identified an increased risk of CMV‐DNAemia in older recipients, as well as in patients with a prolonged postoperative course, indicating that patients with a prolonged initial postoperative course are vulnerable to CMV infections throughout the entire first postoperative year. However, in our multivariate model, the proposed effect of the recipient's age was small. In contrast, D+/R− and D−/R+ constellations were shown to be independent and strong risk factors and these patients may benefit from more individualized prophylaxis. Even if this result appears unsurprising at first glance, since these are known risk factors for CMV infections, it is a new finding that this risk continues to exist despite regular CMV prophylaxis calling for more individualized therapy including, prolonged or repeated CMV prophylaxis with antiviral drugs and CMV immunoglobulins.

## AUTHOR CONTRIBUTIONS

**Moritz Benjamin Immohr**: Conceptualization; data curation; formal analysis; investigation; methodology; project administration; validation; visualization; writing—original draft. **Daniel Oehler**: Data curation; validation; writing—review and editing. **Freya Sophie Jenkins**: Data curation; validation; writing—review and editing. **Nikolas Kalampokas**: Data curation; validation; writing—review and editing. **Vincent Hendrik Hettlich**: Data curation; validation; writing—review and editing. **Dennis Sigetti**: Data curation; validation; writing—review and editing. **Fabian Voß**: Data curation; validation; writing—review and editing. **Hannan Dalyanoglu**: Data curation; validation; writing—review and editing. **Hug Aubin**: Data curation; validation; writing—review and editing. **Payam Akhyari**: Data curation; resources; supervision; validation; writing—review and editing. **Artur Lichtenberg**: Project administration; resources; supervision; validation; writing—review and editing. **Udo Boeken**: Conceptualization; data curation; project administration; resources; supervision; validation; writing—review and editing.

## CONFLICT OF INTEREST STATEMENT

The authors declare no conflict of interest.

## ETHICS STATEMENT

The study was approved by the University's Local Ethics Committee, in accordance with the principles of the Declaration of Helsinki (local study ID: 4567, approved January 31, 2014). Before inclusion, written informed consent for the scientific use of anonymized patient data was obtained from all patients.

## Data Availability

The data underlying this article will be shared on reasonable request to the corresponding author.
